# SomatiX: accurate and efficient somatic SNV detection from matched tumor-normal long-read DNA sequencing

**DOI:** 10.1093/bioinformatics/btag792

**Published:** 2026-10-24

**Authors:** Zelin Liu

**Affiliations:** Independent Researcher, Philadelphia, United States

## Abstract

**Motivation:**

Accurate somatic single-nucleotide variant (SNV) calling from matched tumor-normal long-read DNA sequencing remains challenging because sequencing errors, variable tumor purity, tumor-in-normal contamination, and low variant allele fractions (VAFs) can obscure tumor-specific signals. Although recent deep-learning methods have improved long-read somatic variant calling, robust low-VAF detection and efficient genome-scale analysis remain important methodological challenges.

**Results:**

We present SomatiX, a deep-learning method for somatic SNV detection from matched tumor-normal long-read DNA sequencing data, built on an allele-aware pileup representation. In comprehensive cancer benchmark analyses across diverse cell-line datasets, sequencing contexts, VAF bins, and minimum variant-supporting read thresholds, SomatiX outperformed DeepSomatic and ClairS in F1 score across most Oxford Nanopore Technologies (ONT) benchmark conditions and achieved competitive performance on Pacific Biosciences (PacBio) data. Across the held-out chromosome 1 cancer cell-line benchmarks, at the threshold of ≥3 variant-supporting reads and a VAF ≥0.05, SomatiX achieved mean F1 scores of 0.986 for ONT data and 0.988 for PacBio data. SomatiX showed particularly strong performance for low-VAF SNVs in ONT data while using a compact model and achieving the fastest runtime among the evaluated tools.

**Availability and implementation:**

SomatiX is available at https://github.com/colinliuzelin/SomatiX and archived at Zenodo under DOI: https://doi.org/10.5281/zenodo.22062438. The repository includes source code, trained models, and benchmarking scripts.

## 1 Introduction

Long-read DNA sequencing offers major advantages for cancer genome analysis by improving the detection of structural variants, resolving complex genomic regions, and preserving long-range variant linkage (Wenger *et al.* 2019, Logsdon *et al.* 2020, De Coster *et al.* 2021). Advances in Oxford Nanopore Technologies (ONT) and Pacific Biosciences (PacBio) sequencing, together with improved computational analysis, have expanded the use of long-read sequencing for comprehensive genome characterization. Recent deep-learning-based methods have enabled highly accurate germline small-variant detection from ONT and PacBio data, with performance now matching or exceeding that of short-read methods (Poplin *et al.* 2018, Shafin *et al.* 2021, Zheng *et al.* 2022). However, accurate somatic SNV detection from matched tumor-normal long-read sequencing remains more challenging than germline SNV calling.

Somatic SNV calling is difficult because true tumor-derived allele signals must be distinguished from sequencing errors, germline variants, and allele evidence present in the matched normal sample. This problem is especially challenging in samples with low tumor purity or tumor-in-normal (TIN) contamination, where tumor-specific allele signals are diluted and the contrast between tumor and normal samples is weakened (Cibulskis *et al.* 2013, Taylor-Weiner *et al.* 2018, Mitchell *et al.* 2024, Park *et al.* 2026). Low variant allele fraction (VAF) mutations further complicate detection, because subclonal architecture, copy-number changes, and tumor heterogeneity can produce weak alternate-allele signals that overlap with long-read error profiles (Dentro *et al.* 2021). Together, these challenges limit the broader application of long-read sequencing to somatic SNV detection in cancer genomes.

The development of DeepSomatic and ClairS has demonstrated the potential of deep-learning-based approaches for long-read somatic variant calling (Park *et al.* 2026, Zheng *et al.* 2026). DeepSomatic extends the DeepVariant framework by converting aligned tumor-normal reads into pileup image tensors and classifying candidate variants with a convolutional neural network. ClairS builds on the Clair framework for somatic small-variant calling from matched tumor-normal long-read data. Both methods have shown promising performance in ONT and PacBio data, and DeepSomatic further established multi-cancer benchmark resources with high-quality somatic variant sets from multiple tumor-normal cancer cell-line pairs. These resources enable systematic benchmarking across cancer backgrounds and sequencing conditions. Nevertheless, robust detection of low-VAF somatic SNVs remains difficult, particularly when weak tumor allele signals must be separated from sequencing errors and normal-sample contamination. At the same time, genome-scale tumor-normal analysis remains computationally demanding.

In this study, we present SomatiX, a deep-learning method for somatic SNV detection from matched tumor-normal ONT and PacBio long-read DNA sequencing data. SomatiX uses an allele-aware pileup representation to capture tumor and normal allele-support patterns around candidate variants, enabling accurate discrimination of somatic SNVs from germline variants and sequencing artifacts. Across multiple cancer cell-line benchmarks, SomatiX improved somatic SNV calling performance, with particularly strong gains for low-VAF variants in ONT data, while achieving the fastest runtime among the evaluated tools.

## 2 Materials and methods

### 2.1 Sequencing and benchmark datasets

This study used publicly available matched tumor-normal whole-genome sequencing datasets generated with ONT R10 and PacBio HiFi sequencing. The cancer cell-line panel included five tumor-normal pairs used for model development: HCC1395, H1437, HCC1937, HCC1954, and H2009 (Park *et al.* 2026). For model training and validation, aligned files in Binary Alignment/Map (BAM) format and benchmark files for these cancer cell lines, including truth annotations for both germline and somatic variants, were obtained from the DeepSomatic Google Cloud repository. In addition, the GIAB HG008 (McDaniel *et al.* 2025) matched tumor-normal cancer sample was used as an independent external test set and was not used for model training, validation or data augmentation. For final HCC1395 testing, somatic truth calls and high-confidence regions were obtained from the SEQC2 benchmark resource (Zhao *et al.* 2021). HG008 BAM files and benchmark resources were obtained from the GIAB Cancer Genome in a Bottle resource. All BAM and benchmark analyses were performed against the GRCh38 reference genome. Links to all input files and benchmark resources are provided in Table 1, available as supplementary data at *Bioinformatics* online.

### 2.2 Chromosome split for model training

To avoid overlap between model fitting, validation, and final testing, chromosome 1 was held out as the test chromosome for each cancer cell line. Chromosomes 2–20 were used for training, and chromosomes 21 and 22 were used for validation and checkpoint selection. The same chromosome split was applied to both raw and augmented datasets. HG008 was evaluated only as an external raw test sample and was not included in the chromosome-based training or validation split.

### 2.3 Coverage and purity augmentation for training and testing

For HCC1395, H1437, HCC1937, HCC1954, and H2009 cancer cell lines, the raw matched tumor-normal BAM pair was analyzed together with eight augmented tumor-normal conditions. Augmented BAMs were generated from the original aligned BAMs by read downsampling and tumor-normal read mixing. Tumor and normal reads were downsampled independently using fixed random seeds, and mixed BAMs were produced by merging the selected tumor and normal reads. The same augmentation definitions were used for ONT and PacBio HiFi when matched BAM depth allowed generation of the target condition.

The coverage-only conditions used pure tumor and pure normal BAMs at four tumor-normal coverage combinations: 60×/30×, 30×/30×, 60×/20×, and 30×/20×.

The reduced tumor-purity conditions fixed the total mixed tumor-sample depth at 60× and generated tumor BAMs by mixing tumor and matched normal reads, which were then compared against a pure 30× normal-control BAM. Two tumor-purity conditions were evaluated: 80% tumor purity, consisting of 48× tumor plus 12× normal reads, and 60% tumor purity, consisting of 36× tumor plus 24× normal reads.

The normal-contamination conditions evaluated a pure 60× tumor BAM against a contaminated normal-control BAM with a nominal total depth of 30×. The contaminated normal-control BAMs were generated by mixing matched normal and tumor reads at two ratios: 95% normal plus 5% tumor reads and 90% normal plus 10% tumor reads.

When available normal coverage was lower than the nominal target, all available normal reads were retained and the contaminating tumor depth was rescaled to preserve the intended tumor-normal fraction.

### 2.4 Candidate SNV selection for SomatiX

SomatiX first selected candidate SNV sites from the tumor BAM. Unless otherwise specified, candidates required at least three reads supporting the alternate allele (ALT), a VAF of at least 0.05, a minimum read mapping quality of 20, and a minimum base quality of 10. Candidate generation was restricted to single-nucleotide substitutions. For training and benchmarking, candidates outside the matched high-confidence benchmark regions were excluded.

### 2.5 Allele-aware feature representation

For each candidate SNV, SomatiX extracted paired tumor and normal features from a 61-bp window centered on the candidate position. Reads overlapping the candidate site were assigned according to the allele observed at the candidate position and read orientation, producing four allele-strand groups: (i) REF_FWD: forward-strand reads supporting the reference allele, (ii) REF_REV: reverse-strand reads supporting the reference allele, (iii) ALT_FWD: forward-strand reads supporting the alternate allele, and (iv) ALT_REV: reverse-strand reads supporting the alternate allele.

The following feature groups were generated for both tumor and normal samples:

1. Reference sequence: a 4 × 61 one-hot matrix for A, C, G, and T in the local reference window.
2. Allele-strand base fractions: four 6 × 61 matrices, one for each allele-strand group, with rows corresponding to A, C, G, T, deletion, and insertion. The sequence context and four allele-strand base-fraction matrices form a five-channel tensor per sample, conceptually 5 × 6 × 61 after padding the sequence channel to match the six base-fraction rows.
3. Allele-strand coverage profiles: four length-61 vectors normalized by the maximum local allele-strand coverage for the candidate.
4. Allele-strand base-quality profiles: four length-61 vectors of mean base quality, normalized by a Phred quality score of 40 and capped at 1.
5. Allele-strand mapping quality: four scalar mean mapping-quality values normalized by 60.
6. Allele-strand mismatch rate: four scalar values representing mean read-level mismatch rate for the corresponding allele-strand groups.
7. Candidate-site total coverage: one scalar representing total read depth at the candidate position, normalized by 100 and capped at 1.

Tumor and normal samples used the same allele-strand feature structure and were processed as paired inputs.

### 2.6 Training label assignment for candidate SNV classification

Candidate labels were assigned using the matched truth VCF and high-confidence BED. PASS somatic SNVs in the truth VCF were labeled as somatic. Germline records were labeled from genotype information as heterozygous or homozygous germline. Candidate SNVs located within high-confidence regions but absent from the truth VCF were labeled as reference candidates. SomatiX was therefore trained as a four-class classifier to distinguish reference candidates, heterozygous germline SNVs, homozygous germline SNVs, and somatic SNVs.

### 2.7 Deep-learning model training

SomatiX was implemented using PyTorch (v2.12.0) with CUDA (v12.8). SomatiX used separate tumor and normal network branches with the same feature definitions and architecture. In each branch, the combined sequence/base-fraction tensor was processed by convolutional neural network (CNN) layers, while coverage, base-quality, mapping-quality, mismatch-rate, and candidate-site total coverage features were processed by fully connected layers. The tumor and normal branch outputs were then concatenated and passed through additional fully connected layers followed by a four-class output layer. For each candidate SNV, the model produced class probabilities using a softmax function over four classes: reference candidate, heterozygous germline SNV, homozygous germline SNV, and somatic SNV. The predicted class was assigned as the class with the highest probability. Detailed network architecture parameters are provided in Tables 2 and 3, available as supplementary data at *Bioinformatics* online.

Models were first trained for 30 epochs using cross-entropy loss and the Adam optimizer with a learning rate of 0.001, a weight decay of 1 × 10^−5^, a batch size of 50 000, and a random seed of 2026. The HCC1395 model was trained using the HCC1395 raw dataset and eight augmented datasets, as described above, with checkpoints selected based on the best validation somatic F1 score. The SomatiX multi-cancer model was subsequently fine-tuned from the HCC1395 model using a lower learning rate of 0.0001. Fine-tuning used HCC1395 raw and eight augmented datasets, plus raw datasets from HCC1937, HCC1954, H1437, and H2009. The final multi-cancer checkpoint was selected based on the best validation somatic F1 score across 10 epochs. Both models used the same chromosome split, candidate filters, feature representation, and model architecture.

### 2.8 Use of DeepSomatic for somatic variant calling

DeepSomatic v1.10.0 was used as a comparator somatic variant caller in tumor-normal mode. For each dataset, DeepSomatic was run with the matched tumor BAM, matched normal BAM, GRCh38 reference genome, and chromosome restriction applied in the benchmark analysis. ONT datasets were analyzed with “--model_type=ONT” using the default settings, and PacBio HiFi datasets were analyzed with “--model_type=PACBIO” using the default settings.

### 2.9 Use of ClairS for somatic variant calling

ClairS v0.4.5 was used as a second comparator somatic variant caller in paired tumor-normal mode. ClairS was run with the matched tumor BAM, matched normal BAM, GRCh38 reference genome, chromosome restriction, and platform-specific model. ONT datasets were analyzed with “--platform ont_r10_dorado_sup_5khz_ssrs”. PacBio HiFi datasets were analyzed with the PacBio HiFi model using “--platform hifi_revio_ssrs” for Revio data. For both ONT and PacBio HiFi analyses, ClairS was run with “--min_coverage 3” so that low-depth SNV sites with at least three ALT reads and VAF ≥0.05 were not removed by the default ClairS coverage filter.

### 2.10 Benchmarking of somatic SNV calls

Variant calls from SomatiX, DeepSomatic, and ClairS were benchmarked using som.py (v0.3.8) from hap.py with the matched truth VCF, high-confidence BED file, and GRCh38 reference genome (Krusche *et al.* 2019). Benchmarking was restricted to genomic regions defined by the matched high-confidence BED file. The som.py feature-table output was used for downstream summary analyses. Only SNV rows were retained. Downstream metric calculation was further restricted to sites with at least three ALT reads and VAF ≥0.05, calculated from reads with mapping quality ≥20 and base quality ≥10. true-positive (TP), false-positive (FP), and false-negative (FN) counts were taken from the filtered som.py result tags, and precision, recall, and F1 scores were recalculated from these counts.

### 2.11 ALT-read threshold and VAF-bin analyses

For ALT-read threshold analysis, the minimum ALT-read threshold was varied at ≥3, ≥6, ≥9, and ≥12 while VAF was fixed at ≥0.05. For VAF-bin analysis, sites were required to have at least three ALT reads and were grouped into VAF bins of 0.05–0.20, 0.20–0.35, 0.35–0.50, and 0.50–1.00.

### 2.12 Permutation-based feature-importance analysis

Permutation-based feature-importance analysis was performed to assess the contribution of each feature group to SomatiX predictions. For each feature group, feature values were randomly permuted across candidate sites in the test set while all other input features were kept unchanged. Somatic SNV prediction was then repeated using the permuted features, and performance was evaluated by F1-score. Feature importance was quantified as the decrease in F1-score relative to the unpermuted baseline. Larger F1-score decreases indicated greater contribution of the corresponding feature group to model performance. The analysis was performed separately for the ONT and PacBio HiFi models.

### 2.13 Runtime and memory measurement

Runtime and peak memory usage were measured for SomatiX, DeepSomatic, and ClairS using HCC1395 chromosome 1 and whole-genome datasets with 60× tumor and 30× normal coverage. Whole-genome runtime was measured by sequentially processing chromosomes 1–22. All tools were run with 24 threads on a desktop workstation equipped with an Intel Core Ultra 9 285K CPU and an NVMe SSD. Input, output, and intermediate files were stored on the NVMe SSD during benchmarking. For SomatiX, runtime and memory measurements included the complete calling workflow, including candidate selection, feature extraction, and prediction.

## 3 Results

### 3.1 SomatiX uses an allele-aware tumor-normal representation for long-read somatic SNV detection

We developed SomatiX to detect somatic SNVs from matched tumor-normal ONT and PacBio long-read DNA sequencing data. Somatic SNV calling requires distinguishing weak tumor-specific allele evidence from germline variation and long-read sequencing artifacts. To address this challenge, SomatiX first identifies candidate sites from tumor reads and then extracts matched tumor and normal pileup-derived features within a 61-nt window centered on each candidate site (Fig. 1A).

**Figure 1:**
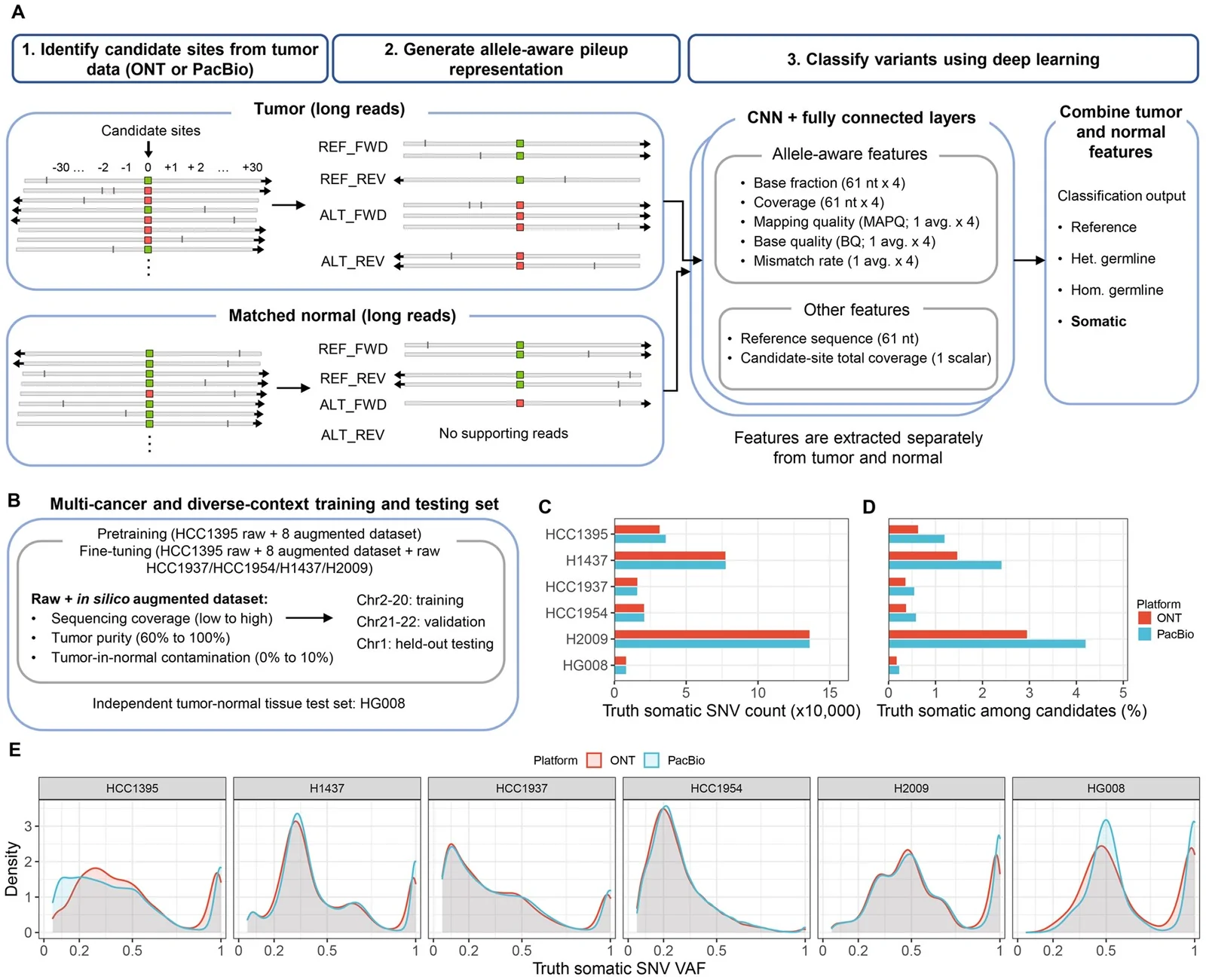
SomatiX workflow and benchmark design. (A) SomatiX identifies candidate sites from tumor long reads, extracts matched tumor-normal allele-aware pileup features in a 61-nt window, and classifies candidates as reference, heterozygous germline, homozygous germline, or somatic SNV. (B) Two-step training strategy using HCC1395 raw and *in silico* augmented datasets for pretraining, followed by multi-cancer fine-tuning with additional raw HCC1937, HCC1954, H1437, and H2009 datasets. Chromosomes 2–20, 21–22, and 1 were used for training, validation, and held-out testing, respectively. HG008 was used as an independent benchmark dataset. (C and D) Truth somatic SNV counts (C) and percentages of somatic SNVs among total candidates (D) across ONT and PacBio datasets, where candidates were defined as sites with at least three ALT-supporting tumor reads and tumor VAF ≥0.05. (E) Truth somatic SNV VAF distributions across evaluated datasets for sites with tumor VAF ≥0.05.

The central methodological design of SomatiX is its allele-aware tumor-normal representation. Rather than constructing features from a common pileup of reads carrying different alleles, SomatiX first partitions reads according to candidate REF/ALT support and strand orientation into REF_FWD, REF_REV, ALT_FWD, and ALT_REV groups. Sequencing features are then summarized separately within each allele-strand group for both the tumor and matched-normal samples. This design preserves information directly relevant to somatic SNV calling, including whether the alternate allele is enriched in the tumor, absent or depleted in the matched normal, and supported consistently across both strands. For each allele-strand group, SomatiX summarizes base fraction, coverage, mapping quality, base quality, and mismatch rate. SomatiX further incorporates the 61-nt reference sequence context and candidate-site total coverage, allowing the model to learn local sequence-dependent error patterns together with REF/ALT read-support patterns in tumor and matched-normal samples. The principal differences in input representation among SomatiX, DeepSomatic, and ClairS are summarized in Table 4, available as supplementary data at *Bioinformatics* online.

SomatiX uses paired tumor and normal network branches, each combining convolutional and fully connected layers to learn sample-level representations, which are then integrated for final four-class classification: reference, heterozygous germline SNV, homozygous germline SNV, or somatic SNV. This formulation explicitly models the major candidate categories encountered during tumor-normal variant calling rather than treating somatic detection as a binary classification problem.

To evaluate and improve generalizability across cancer backgrounds, we trained SomatiX using a two-step strategy based on five tumor-normal cancer cell-line pairs: HCC1395, HCC1937, HCC1954, H1437, and H2009 (Fig. 1B). We first trained an HCC1395 model using the HCC1395 raw dataset and eight *in silico* augmented datasets, generating nine HCC1395 training conditions in total. The augmented datasets included four coverage-downsampled conditions with different tumor-normal coverage combinations (60×/30×, 60×/20×, 30×/30×, and 30×/20×), two tumor-purity mixtures with 80% and 60% tumor purity, and two TIN contamination mixtures, in which 5% or 10% of the normal-sample reads were replaced by tumor-derived reads. Starting from this model, we then fine-tuned a multi-cancer SomatiX model using the HCC1395 raw and augmented datasets together with additional raw datasets from HCC1937, HCC1954, H1437, and H2009. Together, these conditions exposed the model to variation across cancer backgrounds, sequencing coverage, tumor purity, and TIN contamination. For each cell-line pair, chromosome 1 was held out from model development and used for testing, enabling evaluation on genomic regions not seen during training. To further assess generalization beyond the training cell-line systems, we evaluated SomatiX on the independent HG008 tumor-normal benchmark dataset.

Candidate sites showed strong class imbalance, with somatic SNVs representing only a small fraction of all candidates, approximately 1%–4% across datasets and platforms (Fig. 1C and D). This imbalance highlights a central challenge for model training and evaluation: somatic mutations must be separated from a much larger background of sequencing artifacts and germline variants. The evaluated benchmark datasets also varied in truth somatic SNV counts and VAF distributions (Fig. 1C and E), providing a diverse resource for training and testing SomatiX across cancer backgrounds and sequencing platforms.

### 3.2 SomatiX improves somatic SNV detection across tumor-normal long-read cancer benchmarks

We next benchmarked SomatiX against DeepSomatic and ClairS using held-out chromosome 1 test sets from the cancer cell-line benchmarks, evaluating ONT and PacBio data separately. Across minimum ALT-read thresholds, the SomatiX multi-cancer model achieved consistently high F1 scores and showed its strongest advantage in ONT data (Fig. 2A and B; Table 5, available as supplementary data at *Bioinformatics* online). This improvement was most pronounced at the ≥3 ALT-read threshold, where variant-supporting evidence is weakest and somatic SNVs are most difficult to distinguish from sequencing errors. In PacBio data, SomatiX achieved F1 scores comparable to DeepSomatic across all evaluated ALT-read thresholds and generally higher than ClairS.

**Figure 2:**
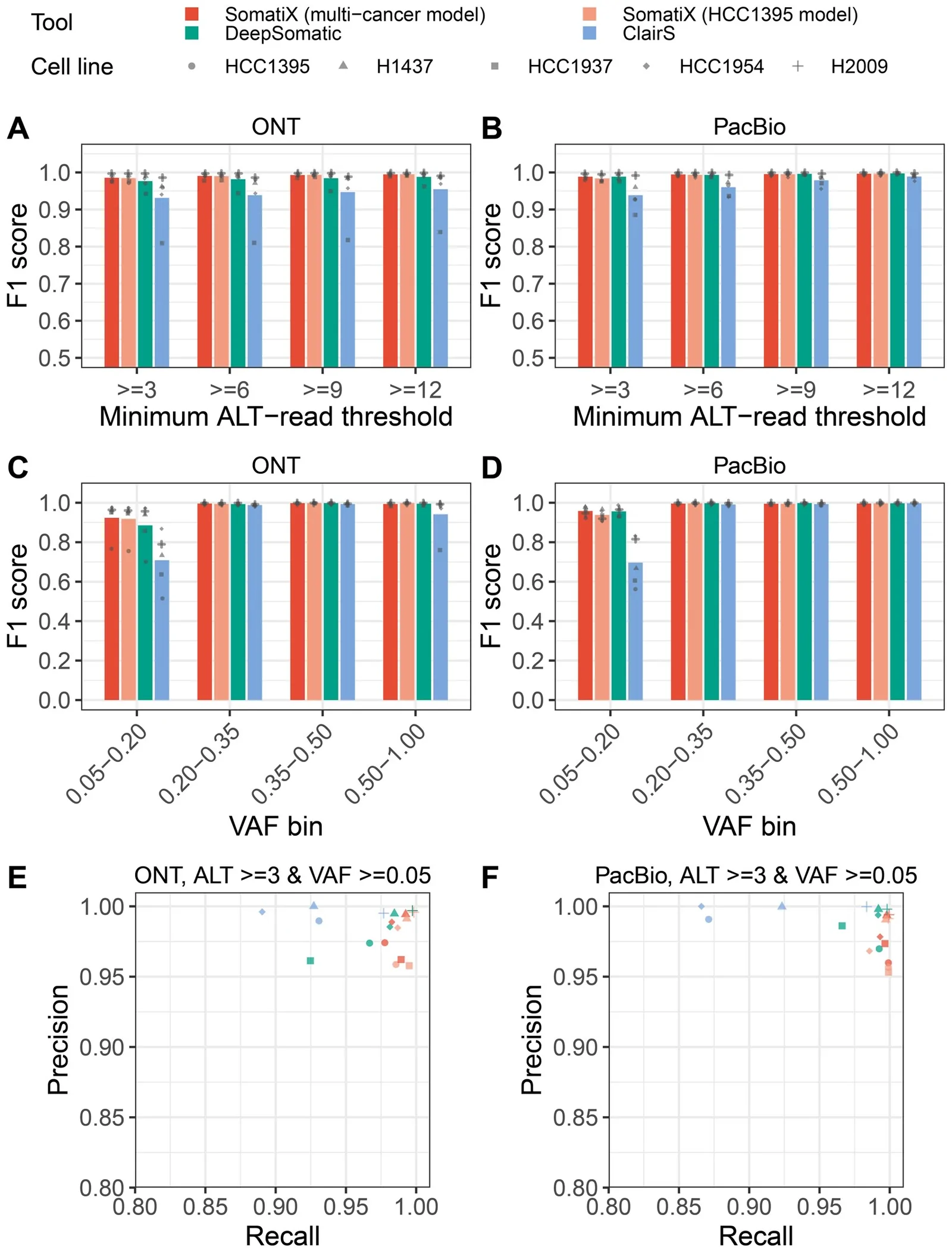
Benchmarking SomatiX across held-out chromosome 1 raw tumor-normal cancer datasets. (A and B) F1 score across minimum ALT-read thresholds for ONT and PacBio raw datasets, respectively. Bars represent mean performance across evaluated cell-line datasets, and points represent individual cell lines. (C and D) F1 score across VAF bins for ONT and PacBio raw datasets, respectively, with at least 3 ALT-supporting reads. (E and F) Precision-recall comparison for ONT and PacBio raw datasets using sites with ≥3 ALT reads and VAF ≥0.05. SomatiX multi-cancer model, SomatiX HCC1395 model, DeepSomatic, and ClairS were evaluated on held-out chromosome 1 using the same benchmark regions and post-filtering criteria.

The advantage of SomatiX was particularly evident for low-VAF somatic SNVs within the predefined 0.05–0.20 VAF range. When variants were stratified by VAF, the 0.05–0.20 bin was the most challenging category for all methods (Fig. 2C and D; Table 6, available as supplementary data at *Bioinformatics* online). In this low-VAF range, SomatiX showed the clearest improvement in ONT data. ClairS showed particularly low F1 scores in the 0.05–0.20 VAF bin for several benchmark datasets, reflecting reduced recall despite high precision for low-VAF somatic SNVs. At higher VAF bins, SomatiX led or matched the best-performing methods across both sequencing platforms. At the standard benchmarking threshold of ≥3 ALT-supporting reads and VAF ≥0.05, SomatiX achieved mean F1 scores of 0.986 for ONT data and 0.988 for PacBio data (Table 7, available as supplementary data at *Bioinformatics* online).

Precision-recall analysis showed that the F1-score improvements reflected a favorable balance between precision and recall rather than a gain in one metric at the expense of the other (Fig. 2E and F). In ONT data, SomatiX generally occupied the high-precision and high-recall region relative to DeepSomatic and ClairS. We also noted that ClairS showed a conservative calling pattern across the tested samples, maintaining high precision but often with reduced recall. In PacBio data, SomatiX achieved precision-recall performance comparable to DeepSomatic and generally higher than ClairS.

We also compared the SomatiX multi-cancer model with the HCC1395-only model to assess the effect of fine-tuning on additional cancer cell-line datasets. The multi-cancer model achieved performance comparable to or higher than the HCC1395-only model across most ONT and PacBio benchmarks, with the clearest improvement observed for low-VAF somatic SNVs (Fig. 2C and D).

To further assess sample-level generalization, we applied the fixed HCC1395-only model to chromosomes 1–22 of HCC1937, HCC1954, H1437, and H2009, none of which were used for development of this model. The HCC1395-only model maintained high performance across these four unseen tumor-normal cell lines, with mean F1 scores of 0.979 for ONT and 0.985 for PacBio HiFi (Table 8, available as supplementary data at *Bioinformatics* online; H1437 PacBio calculated excluding chromosome 9), and also maintained strong performance in the low-VAF range of 0.05–0.20, with mean F1 scores of 0.930 for ONT and 0.931 for PacBio HiFi (Table 9, available as supplementary data at *Bioinformatics* online). These results demonstrate that SomatiX generalizes beyond held-out genomic regions to tumor-normal samples not represented during model development.

### 3.3 SomatiX maintains robust performance across genomic contexts and identifies key predictive features

We next evaluated SomatiX across genomic contexts that are often challenging for variant calling. Across coding sequences, non-coding regions, GC-extreme regions, tandem repeats, low-mappability regions, and long homopolymers, the SomatiX multi-cancer model maintained high F1 scores in both ONT and PacBio data, with performance varying by genomic context (Fig. 3A and B; Table 10, available as supplementary data at *Bioinformatics* online). The most consistent performance drop was observed in long homopolymers, where all methods showed reduced F1 scores and greater variability across cell lines. In ONT data, SomatiX generally outperformed DeepSomatic and ClairS across most stratified regions. In PacBio data, SomatiX remained comparable to DeepSomatic overall, although DeepSomatic performed slightly better in tandem repeats and low-mappability regions.

**Figure 3:**
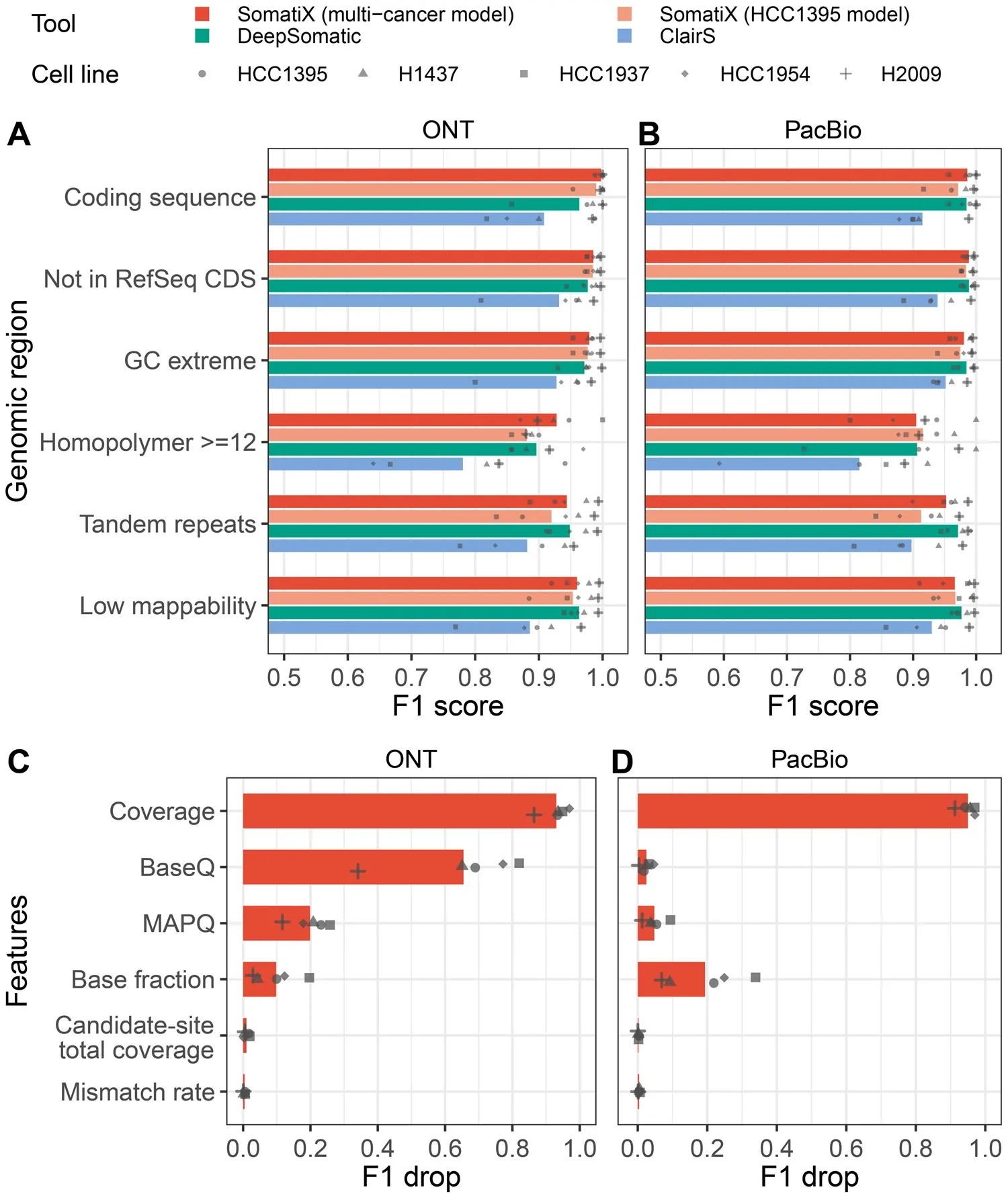
SomatiX performance across genomic contexts and feature groups. (A and B) F1 score across genomic stratification categories for ONT and PacBio datasets, respectively. Bars represent mean values across evaluated cell-line datasets, and points represent individual cell lines. Stratified regions include coding sequence, non-RefSeq coding regions, GC-extreme regions, long homopolymers, tandem repeats, and low-mappability regions. (C and D) Permutation-based feature importance for ONT and PacBio models, measured as the decrease in F1 score after permuting each feature group. Evaluated feature groups include coverage, base quality (BaseQ), mapping quality (MAPQ), base fraction, candidate-site total coverage, and mismatch rate. All analyses used SNV sites with ≥3 ALT reads and VAF ≥0.05 within high-confidence benchmark regions.

We then performed permutation-based feature-importance analysis to assess which feature groups contributed most to SomatiX predictions. Permuting coverage features caused the largest F1-score decrease in both ONT and PacBio models, indicating that coverage was the dominant predictive feature group (Fig. 3C and D). In ONT data, base-quality, mapping-quality, and base-fraction features also contributed to performance, consistent with the greater importance of sequencing-error-associated features for ONT variant calling. In PacBio data, base-fraction features showed a relatively larger contribution than base-quality and mapping-quality features, although coverage remained the dominant feature group. Candidate-site total coverage and mismatch-rate features had limited effects in both platforms. These platform-specific differences are consistent with the lower overall sequencing error rate of PacBio HiFi reads compared with ONT reads, which may reduce the relative contribution of error-associated features such as base quality and mapping quality.

### 3.4 SomatiX maintains robust performance across coverage, tumor purity, and TIN contamination

We next evaluated SomatiX under *in silico* mixture conditions designed to test robustness to sequencing depth, tumor purity, and TIN contamination. Across tumor-normal coverage combinations, the SomatiX multi-cancer model maintained high F1 scores in both ONT and PacBio data, including reduced-depth settings with 30× tumor and/or 20× normal coverage (Fig. 4A and B; Table 11, available as supplementary data at *Bioinformatics* online). In ONT data, SomatiX generally outperformed DeepSomatic and ClairS across coverage conditions. In PacBio data, SomatiX achieved F1 scores comparable to DeepSomatic and generally higher than ClairS.

**Figure 4:**
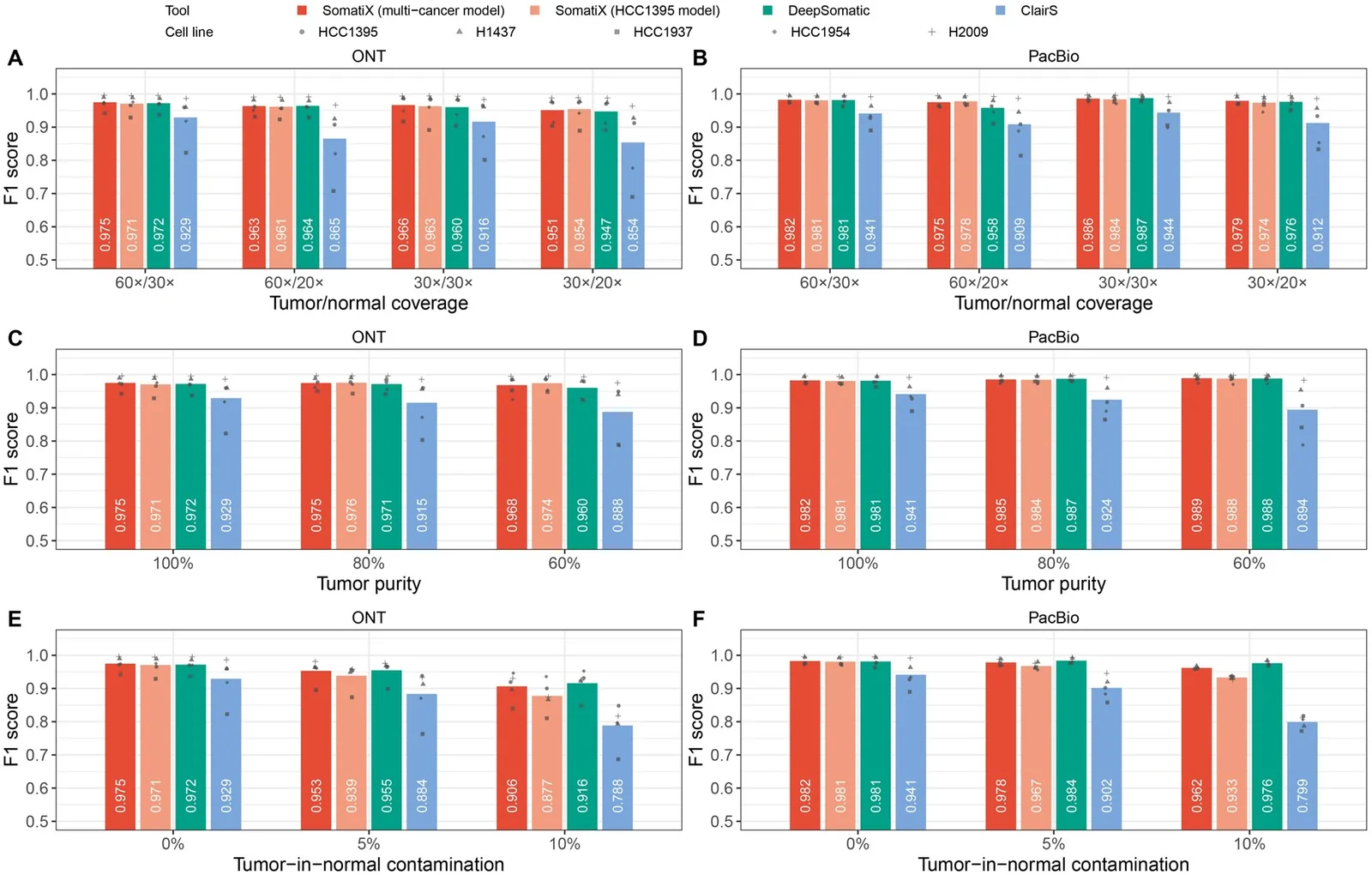
SomatiX performance across *in silico* mixture conditions. (A and B) F1 score across tumor-normal coverage conditions for ONT and PacBio datasets, respectively. (C and D) F1 score across tumor-purity conditions. (E and F) F1 score across TIN contamination levels. Bars represent mean values across evaluated cell-line datasets, and points represent individual cell lines. All analyses used SNV sites with ≥3 ALT reads and VAF ≥0.05 within high-confidence benchmark regions.

Reduced tumor purity had a limited effect on SomatiX performance. When tumor purity was decreased from 100% to 80% or 60%, SomatiX maintained high F1 scores in both ONT and PacBio data (Fig. 4C and D). By contrast, TIN was the most challenging mixture condition. At 5% and especially 10% TIN, all methods showed reduced performance because tumor-derived ALT reads in the normal sample weaken the tumor-specific signal (Fig. 4E and F). Nevertheless, SomatiX maintained higher F1 scores than ClairS and remained close to DeepSomatic across most TIN conditions. Together, these results show that SomatiX remains robust across coverage, tumor-purity, and TIN contamination conditions.

### 3.5 HG008 benchmarking highlights the difficulty of low-VAF somatic SNV evaluation

We further evaluated SomatiX on the independent HG008 tumor-normal benchmark dataset. Compared with the cancer cell-line benchmarks, both SomatiX and DeepSomatic showed lower overall performance on HG008, whereas ClairS achieved higher F1 scores, particularly in the low-VAF range (Fig. 5A and B; Table 12, available as supplementary data at *Bioinformatics* online). Stratification by VAF bin showed that the reduced F1 scores for SomatiX and DeepSomatic were mainly driven by low precision in the 0.05–0.20 VAF bin, while performance was substantially higher for variants with VAF >0.20. To further characterize these low-VAF false-positive calls, we examined their distribution across GC-extreme regions, homopolymers ≥12 bp, tandem repeats, and low-mappability regions. The genomic-context distribution was broadly similar across VAF bins, with no consistent enrichment of the 0.05–0.20 VAF false-positive calls in these difficult genomic regions (Supplementary Fig. 1, available as supplementary data at *Bioinformatics* online). However, this analysis does not establish whether individual low-VAF calls represent technical false positives or variants not represented in the current benchmark. The true status of these calls cannot be resolved using the current benchmark, and we therefore refer to them conservatively as unconfirmed.

**Figure 5:**
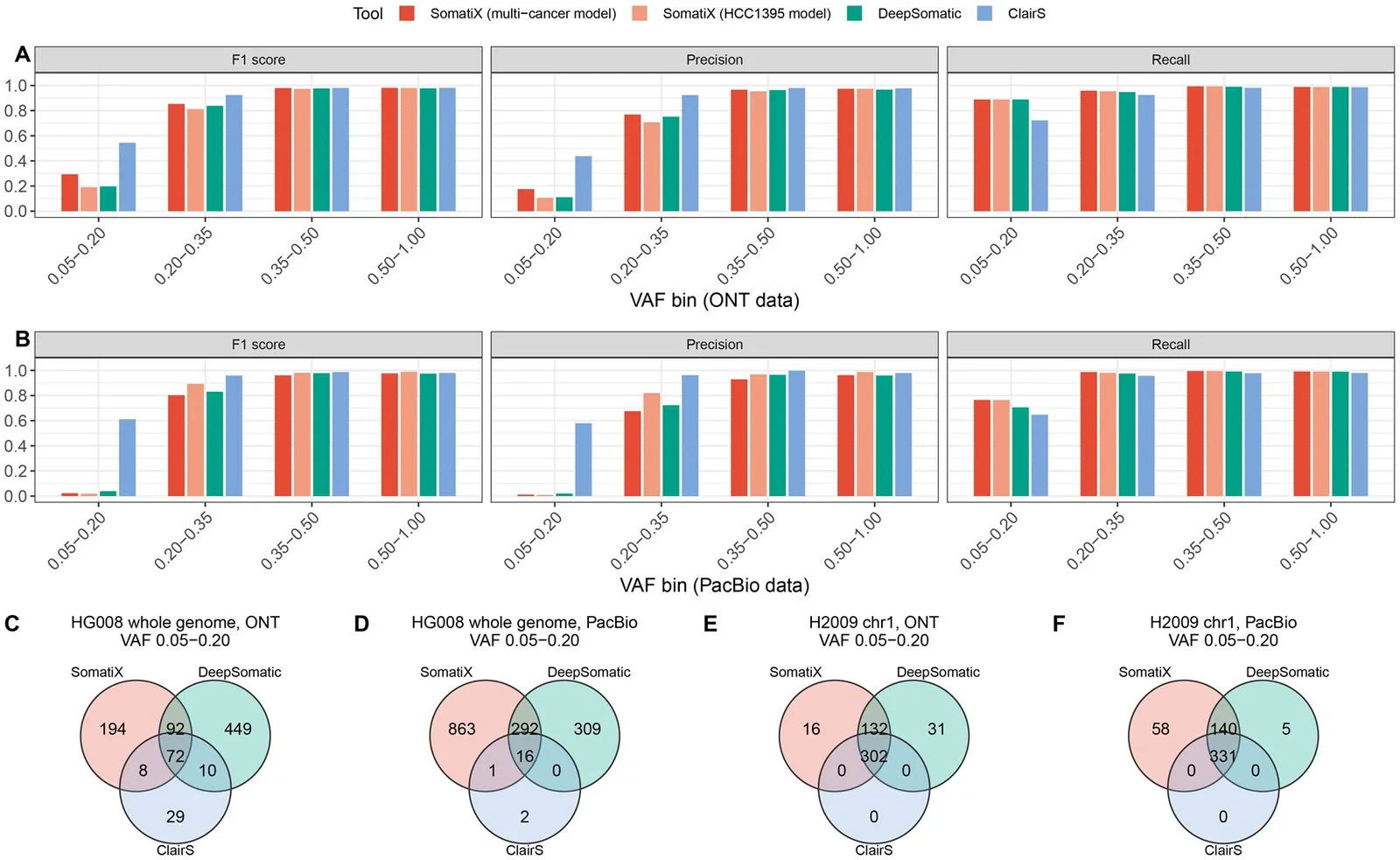
Low-VAF somatic SNV discordance in HG008 benchmark data. (A and B) F1 score, precision, and recall across VAF bins for HG008 ONT and PacBio datasets, respectively. (C and D) Overlap of low-VAF calls detected by SomatiX, DeepSomatic, and ClairS in HG008 ONT and PacBio data. (E and F) Overlap of low-VAF calls in held-out H2009 chromosome 1 ONT and PacBio data. Low-VAF calls were defined as SNVs with a tumor VAF of 0.05–0.20. All analyses used sites with ≥3 ALT reads and VAF ≥0.05 within high-confidence benchmark regions.

One possible explanation is that the HG008 somatic truth set was generated primarily using assembly-based strategies, which are expected to be more effective for higher-VAF clonal variants than for very low-VAF somatic SNVs. Consistent with this possibility, the truth VAF distribution in HG008 was enriched for higher-VAF variants and contained relatively fewer low-VAF variants compared with several cell-line benchmarks (Fig. 1E). Candidate calls in the 0.05–0.20 VAF range may therefore include true tumor-derived signals that are not represented in the current truth set, leading to apparent false positives and reduced precision.

To further examine low-VAF calls, we compared the overlap among SomatiX, DeepSomatic, and ClairS in the 0.05–0.20 VAF bin. In HG008, the overlap among tools was limited, and both SomatiX and DeepSomatic produced many method-specific low-VAF calls in ONT and PacBio data (Fig. 5C and D). This pattern suggests that the apparent low precision in HG008 may partly reflect incomplete representation of low-VAF variants in the current truth set, rather than caller-specific false positives alone. Because H2009 also contained a relatively small fraction of low-VAF truth variants, we used the held-out H2009 chromosome 1 benchmark as a comparison case. In contrast to HG008, SomatiX and DeepSomatic showed much stronger overlap in the same low-VAF range for H2009 (Fig. 5E and F). Overall, these analyses indicate that low-VAF somatic SNV benchmarking remains challenging and should be interpreted carefully, particularly for benchmark datasets with limited representation of low-VAF truth variants.

### 3.6 SomatiX enables fast and memory-efficient somatic SNV detection

We next evaluated the computational efficiency of SomatiX for matched tumor-normal long-read somatic SNV calling. Runtime and peak memory usage were compared with DeepSomatic and ClairS using HCC1395 datasets under the 60× tumor and 30× normal coverage condition, with all tools run using 24 threads on the same desktop workstation.

Using the HCC1395 chromosome 1 benchmark, SomatiX achieved the fastest runtime and lowest peak memory usage among the evaluated tools for both ONT and PacBio data (Fig. 6A and B). For chromosome 1 analysis, SomatiX was 4.5-fold faster than DeepSomatic and 3.8-fold faster than ClairS for ONT data, and 4.7-fold faster than DeepSomatic and 2.8-fold faster than ClairS for PacBio data. SomatiX also used less peak memory, requiring 34.0 GiB for ONT data and 18.7 GiB for PacBio data, compared with 46.6–63.9 GiB for the other tools.

**Figure 6:**
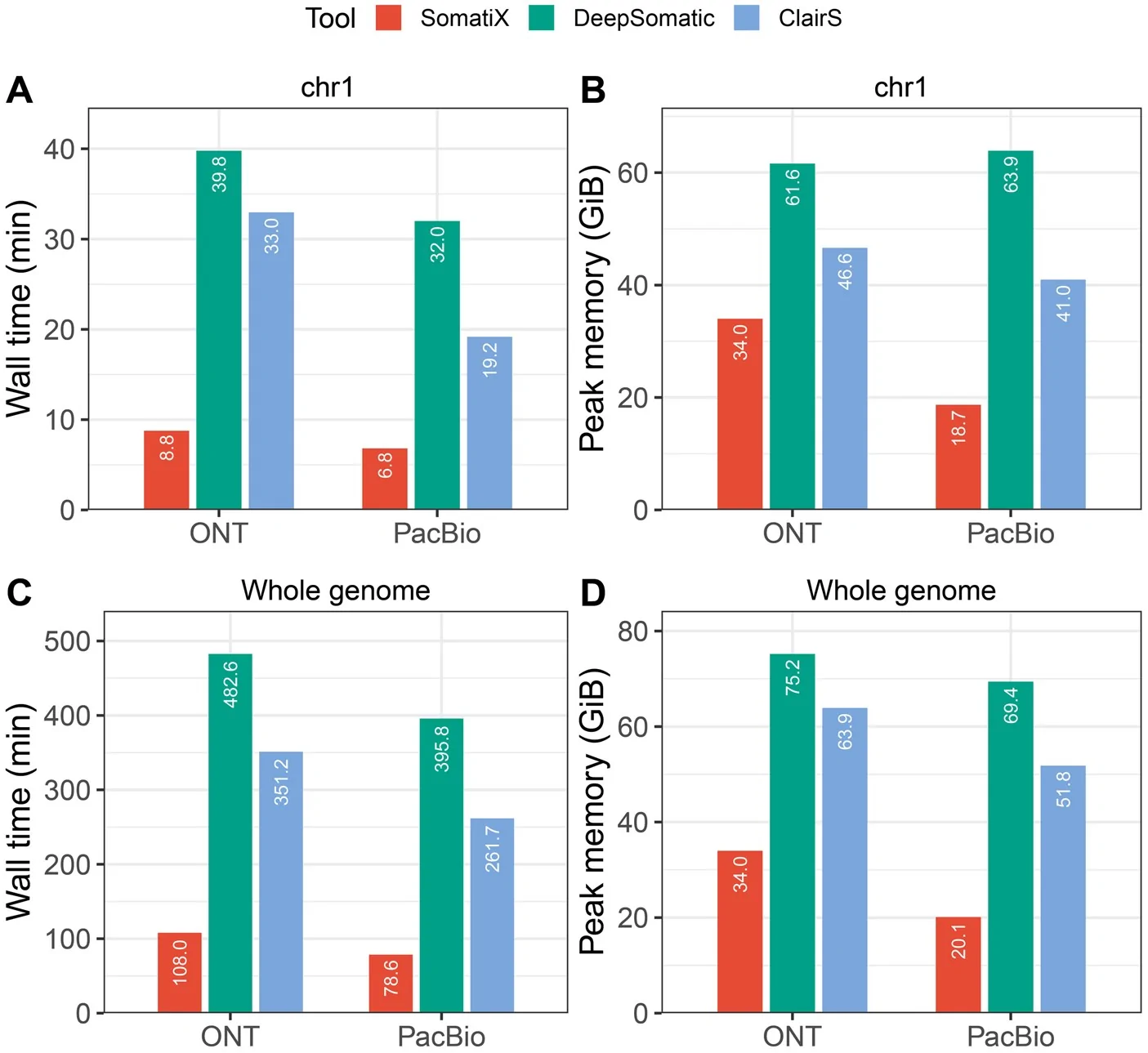
Computational efficiency of SomatiX for long-read somatic SNV detection. Runtime and peak memory usage were measured for SomatiX, DeepSomatic, and ClairS using HCC1395 datasets with 60× tumor and 30× normal coverage. (A and B) Wall-clock runtime and peak memory usage for chromosome 1 analysis. (C and D) Wall-clock runtime and peak memory usage for whole-genome analysis, measured by sequentially processing chromosomes 1–22. Results are shown separately for ONT and PacBio HiFi datasets.

This efficiency advantage was also observed in the whole-genome benchmark, where chromosomes 1–22 were processed sequentially (Fig. 6C and D). SomatiX remained the fastest tool for both ONT and PacBio data and required the lowest peak memory usage among the evaluated tools. Together, these results show that SomatiX provides a computationally efficient framework for matched tumor-normal long-read somatic SNV detection.

## 4 Discussion

Long-read sequencing is increasingly practical for cancer genome analysis, but somatic SNV detection remains a distinct problem from germline variant calling. In germline calling, the expected allele fractions are often close to 0.5 or 1.0, whereas tumor-normal somatic calling must identify weak tumor-specific signals against sequencing errors, germline variation, tumor purity effects, and TIN contamination. This distinction is especially important for low-VAF somatic SNVs, which remain among the most challenging variants for tumor-normal long-read calling. SomatiX addresses this challenge by explicitly representing how reference and alternate alleles are supported across strands in both tumor and matched-normal samples.

The main methodological contribution of SomatiX is therefore not only its neural-network architecture, but its allele-aware tumor-normal representation. By separating REF- and ALT-supporting reads across forward and reverse strands, SomatiX exposes the model to the core evidence used by human and algorithmic somatic callers: enrichment of the alternate allele in the tumor, depletion or absence of the same allele in the matched normal, and balanced support across strands. This design is consistent with the largest gains being observed in low-VAF settings, particularly in ONT data, where weak somatic allele evidence must be distinguished from a higher background of sequencing errors. The feature-importance analysis further supports this interpretation, as coverage-derived allele-support features were the dominant predictive feature group, while error-associated features contributed more strongly in ONT than in PacBio data.

The comparison with DeepSomatic and ClairS also illustrates different trade-offs in long-read somatic variant calling. DeepSomatic uses high-dimensional pileup image tensors and achieved strong performance, especially in PacBio data and some difficult genomic contexts. ClairS builds on the Clair framework for paired tumor-normal long-read somatic small-variant calling but showed weaker performance for low-VAF SNVs in several cell-line benchmarks. SomatiX occupies a complementary design space: it uses a more compact numerical representation focused on allele-support patterns, and this appears sufficient to achieve strong SNV performance while substantially reducing runtime and memory usage. These results suggest that, for somatic SNV detection, carefully structured tumor-normal evidence can provide an effective alternative to larger input representations or larger neural networks.

The benchmark results also emphasize that low-VAF somatic SNV detection is not a single uniform problem. Low tumor purity reduces the tumor allele signal, TIN contamination introduces tumor-derived evidence into the matched normal, and long-read sequencing errors can create low-frequency alternate alleles that resemble real subclonal mutations. SomatiX remained robust across coverage and tumor-purity mixtures, while TIN was the strongest stress condition for all methods. This pattern is expected because TIN directly weakens the contrast between tumor and normal samples, which is the defining signal for somatic calling. These results indicate that future somatic callers should continue to model tumor and normal evidence jointly, rather than optimizing tumor-only sensitivity.

The HG008 analysis highlights a second issue: benchmarking low-VAF somatic variants can be as difficult as calling them. SomatiX and DeepSomatic showed reduced precision in the 0.05–0.20 VAF bin in HG008. The stronger overlap between SomatiX and DeepSomatic in H2009, despite the relatively small fraction of low-VAF truth variants, suggests that the reduced precision observed in HG008 cannot be explained by truth-set composition alone. The reduced low-VAF precision in HG008 may reflect several factors, including germline misclassification, limitations of the current benchmark, sequencing or alignment artifacts, and dataset-specific generalization effects. The available data do not allow these possibilities to be distinguished reliably for individual unconfirmed calls. Because assembly-based truth-set construction is expected to be more complete for higher-VAF clonal variants than for low-VAF subclonal variants, HG008 provides a valuable but still challenging benchmark for evaluating low-frequency somatic SNVs. Future benchmark development may need to combine assembly-based truth sets with orthogonal sequencing, deep targeted validation, and cross-caller concordance to better resolve low-VAF truth.

Computational efficiency is also an important dimension of long-read somatic variant calling. Tumor-normal analysis requires processing two whole-genome long-read datasets, and computational cost can limit scaling to larger cancer cohorts and expanded benchmark studies. By using compact numerical pileup features and a lightweight model with 0.38 million trainable parameters, SomatiX achieved substantially faster runtime and lower memory usage than DeepSomatic and ClairS. This suggests that compact allele-aware representations can provide both predictive accuracy and practical scalability.

SomatiX currently focuses on somatic SNVs and does not call somatic indels. Although indels are important for comprehensive cancer genome analysis, accurate somatic indel detection from ONT and PacBio tumor-normal long-read data remains less mature than somatic SNV detection because indel errors, homopolymer-associated artifacts, and alignment ambiguity are more difficult to model and benchmark.

## 5 Conclusion

SomatiX is a deep-learning method for somatic SNV detection from matched tumor-normal ONT and PacBio long-read DNA sequencing data. By using an allele-aware pileup representation and a compact neural-network architecture, SomatiX improves low-VAF somatic SNV detection while enabling fast and memory-efficient genome-scale analysis. Across cancer benchmark datasets and *in silico* mixture conditions, SomatiX achieved strong performance relative to existing long-read somatic variant callers, with the clearest advantage for low-VAF SNVs in ONT data. SomatiX provides an efficient framework for long-read tumor-normal somatic SNV calling and highlights the need for continued development of robust low-VAF benchmark truth sets.

## Supplementary Material

btag792_Supplementary_Data

## Data Availability

Aligned BAM files and benchmark resources for the cancer cell-line panel, except the final HCC1395 SEQC2 test benchmark, were obtained from the *DeepSomatic Google Cloud repository: gs://brain-genomics-public/publications/park2024_deepsomatic/*. HCC1395 somatic truth calls and high-confidence regions used for final testing were obtained from the SEQC2 benchmark resource: https://sites.google.com/view/seqc2/home/data-analysis/high-confidence-somatic-snv-and-indel-v1-2. HG008 BAM files and benchmark resources were obtained from the GIAB Cancer Genome in a Bottle project: https://www.nist.gov/programs-projects/cancer-genome-bottle. Links to all input BAM files, truth VCFs and high-confidence BED files used in this study are provided in Table 1, available as supplementary data at *Bioinformatics* online. SomatiX source code, trained models, and benchmarking scripts are available at https://github.com/colinliuzelin/SomatiX. The version used in this study is archived at Zenodo under DOI: https://doi.org/10.5281/zenodo.22062438. DeepSomatic is available at https://github.com/google/deepsomatic. ClairS is available at https://github.com/HKU-BAL/ClairS. *hap.py*, including *som.py*, is available at https://github.com/Illumina/hap.py.
