# Supplementary material for: SomatiX: accurate and efficient somatic SNV detection from matched tumor-normal long-read DNA sequencing
Source: Bioinformatics. 2026 Oct 24;42(11):btag792. doi: 10.1093/bioinformatics/btag792 (PMC13725752; doi:10.1093/bioinformatics/btag792)
Supplement: Supplementary file 1 [file btag792_supplementary_data.zip › Supplementary_Figure.docx]

**Supplementary Figure 1.** **Genomic-context distribution of SomatiX false-positive calls across VAF bins in HG008.**

Alt text: Heatmaps showing HG008 SomatiX false-positive counts and percentages across variant allele-fraction bins in GC-extreme, homopolymer, tandem-repeat, and low-mappability regions for ONT and PacBio HiFi data.
SomatiX false-positive calls identified in the HG008 benchmark were stratified by tumor VAF and genomic context for ONT (left) and PacBio HiFi (right). Genomic contexts include GC-extreme regions, homopolymers ≥12 bp, tandem repeats, and low-mappability regions. Each cell shows the number of false-positive calls overlapping the indicated genomic region and, in parentheses, the percentage of all false-positive calls within the corresponding VAF bin. Color intensity represents this percentage. Genomic categories were evaluated independently and are therefore not mutually exclusive.
